# Histopathological image and gene expression pattern analysis for predicting molecular features and prognosis of head and neck squamous cell carcinoma

**DOI:** 10.1002/cam4.3965

**Published:** 2021-05-13

**Authors:** Linyan Chen, Hao Zeng, Mingxuan Zhang, Yuling Luo, Xuelei Ma

**Affiliations:** ^1^ Department of Biotherapy Cancer Center State Key Laboratory of Biotherapy West China Hospital Sichuan University Chengdu China; ^2^ West China School of Medicine West China Hospital Sichuan University Chengdu China

**Keywords:** head and neck cancer, histopathological images, machine learning, transcriptomics

## Abstract

**Background:**

Histopathological image features offer a quantitative measurement of cellular morphology, and probably help for better diagnosis and prognosis in head and neck squamous cell carcinoma (HNSCC).

**Methods:**

We first used histopathological image features and machine‐learning algorithms to predict molecular features of 212 HNSCC patients from The Cancer Genome Atlas (TCGA). Next, we divided TCGA‐HNSCC cohort into training set (*n* = 149) and test set (*n* = 63), and obtained tissue microarrays as an external validation set (*n* = 126). We identified the gene expression profile correlated to image features by bioinformatics analysis.

**Results:**

Histopathological image features combined with random forest may predict five somatic mutations, transcriptional subtypes, and methylation subtypes, with area under curve (AUC) ranging from 0.828 to 0.968. The prediction model based on image features could predict overall survival, with 5‐year AUC of 0.831, 0.782, and 0.751 in training, test, and validation sets. We next established an integrative prognostic model of image features and gene expressions, which obtained better performance in training set (5‐year AUC = 0.860) and test set (5‐year AUC = 0.826). According to histopathological transcriptomics risk score (HTRS) generated by the model, high‐risk and low‐risk patients had different survival in training set (HR = 4.09, *p* < 0.001) and test set (HR=3.08, *p* = 0.019). Multivariate analysis suggested that HTRS was an independent predictor in training set (HR = 5.17, *p* < 0.001). The nomogram combining HTRS and clinical factors had higher net benefit than conventional clinical evaluation.

**Conclusions:**

Histopathological image features provided a promising approach to predict mutations, molecular subtypes, and prognosis of HNSCC. The integration of image features and gene expression data had potential for improving prognosis prediction in HNSCC.

## Graphical abstract

This study extracted histopathological image features of head and neck squamous cell carcinoma, then used image features and machine learning to build prediction models for mutations, molecular subtypes and prognosis. Moreover, we investigated the correlation between image features and gene expressions, and constructed an integrative model to improve survival prediction. Our study indicated the clinical value of histopathological image features in predicting molecular features and prognosis.

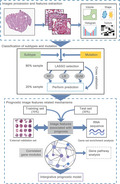

## 1 INTRODUCTION

Head and neck squamous cell carcinoma (HNSCC) is a common heterogeneous cancer of head and neck region, which has over 500,000 new cases every year worldwide.^1^, ^2^ Approximately 40% of patients are diagnosed with early‐stage cancer and usually receive either surgery or radiotherapy, while the majority of patients with locally advanced lesion need multimodality treatments.^2^ These intensive therapies treatments often cause severe acute toxicity (e.g., mucositis and dysphagia), and late organ dysfunction, such as sensorineural deafness, dehisce difficulty, and xerostomia.^3^ Despite the advances in therapeutic techniques, the risk of recurrences or metastatic tumors remains high, leading to a poor prognosis with median survival of 10 months.^4^ In the last decade, immunotherapies and targeted therapies have been applied to improve survival for HNSCC patients.^5^ With the trend of precision medicine modality, it is crucial to identify novel biomarkers that contribute to risk stratification and treatment strategy selection for cancer patients.

The principal risk factors of HNSCC contain exposure to tobacco and alcohol, and human papillomavirus (HPV) infection.^6^ The HPV status can also guide the de‐intensification of therapy in oropharyngeal SCC, because HPV‐positive patients showed better therapeutic response and prognosis while lower risk of relapse and secondary tumors.^7^, ^8^ Except HPV status, other molecular characteristics of HNSCC have not been confirmed. However, many studies about genome sequencing profiling have provided insight into the molecular mechanism and features of HNSCC. Mutations and pathways regarding the cell cycle and survival (*TP53*, *CDKN2A*, *PIK3CA*), *Wnt* signaling (*FAT1*, *AJUBA*), squamous differentiation (*NOTCH1*, *ZNF750*), and chromatin remodeling (*NSD1*, *MLL2*) have been identified.^9^, ^10^ In addition, The Cancer Genome Atlas (TCGA) Network reported several gene expression classes (atypical, mesenchymal, basal, and classical) and methylation subtypes (normal‐like, hypomethylated, hypermethylated, and CpG island hypermethylated) of HNSCC.^9^ These molecular properties have potential in creating opportunities to explore novel biomarkers for diagnosis and prognosis, and assist individualized therapy for patients.^11^ However, no routine genetic tests probably due to expensive cost affect the clinical application and spreading of these genetic and molecular advances. Therefore, there is a need for effective tools to classify the molecular features of HNSCC.

The histopathological examination plays an important role in diagnosis, staging and prognosis of cancer patients. With the advent of computer‐aided images analysis systems, a large amount of histopathological image feature was extracted from digital whole‐slide images, which reflected various characteristics of microscopic morphology of tumor cells and microenvironment.^12^ Previous researches have showed that histopathological image features had great promise in outcome prediction,^12^, ^13^, ^14^ grading and classification of tumors.^15^, ^16^ Furthermore, digital pathology can also serve as a bridge to connect morphological features and omics profiles (genomics, transcriptomics, and proteomics) for better tumor characterization and understanding of underlying biological processes.^17^ Significant correlation between gene mutation, expression, and histopathological image features have been observed in glioblastoma,^16^ lung cancer,^18^ and liver cancer.^19^ In addition, the histopathology‐omics fusion has been previously attempted in several cancers, and achieved a more accurate stratification of prognosis in patients.^18^, ^19^, ^20^, ^21^ Therefore, analyzing the relationship and integration between histopathological and genomic features is an important topic in tumor biology and survival prediction.

Here we focused on the histopathological image analysis and its association with genomics and transcriptomics profiles, which have not been well explored in HNSCC samples before. We first demonstrated the power of histopathological image features in classifying common somatic mutations, transcriptional subtypes, and methylation subtypes of HNSCC. Then we identified the prognostic image features, and assessed the potential correlation between image features, and gene expression patterns by bioinformatics analysis. Subsequently, the integration of histopathological features and transcriptomics data was performed to improve the accuracy of prognosis evaluation for patients with HNSCC.

## 2 MATERIALS AND METHODS

### 2.1 Data source

**FIGURE 1:**
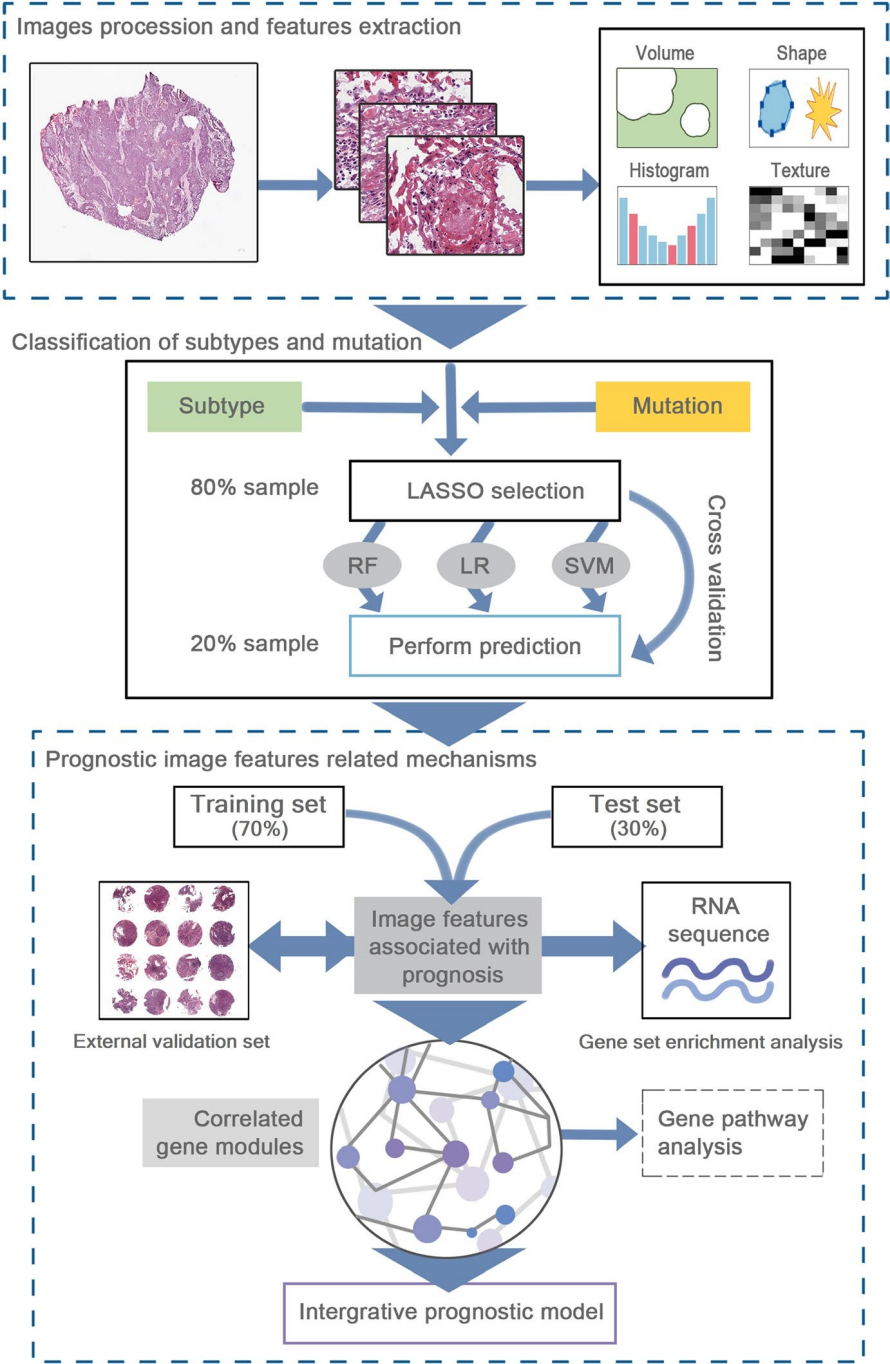
The workflow of data analysis and integration. First, we performed the histopathological image processing and feature extraction. Secondly, three classifiers were constructed by feature selection and 5‐fold cross‐validation, and applied to classify the somatic mutations, transcriptional, and methylation subtypes. Subsequently, we selected the prognostic image features, used bioinformatics analyses to identify correlated gene modules, and established an integrative prognostic model to improve prognosis prediction

The overall framework was summarized in Figure 1. The details of each section were described in following parts. The 212 HNSCC patients with clinical characteristics, somatic mutation, and mRNA sequencing data were acquired from The Cancer Genome Atlas (TCGA) (https://portal.gdc.cancer.gov/). The TCGA cohort was initially diagnosed from 1993 to 2013, and completed follow‐up from 2010 to 2014. We also downloaded the matched H&E‐stained histopathological images (20× or 40× magnification) from The Cancer Imaging Archive (TCIA) (http://www.cancerimagingarchive.net/). In addition, tissue microarrays (TMAs) of 126 HNSCC patients were obtained from Shanghai Outdo Biotech Company (Shanghai, China) and used for external validation. TMA‐HNSCC patients were diagnosed from January 2010 to September 2011, and followed up until March 2017 or death. The ethical approval of TMA cohort was obtained from the National Human Genetic Resources Sharing Service Platform (2005DKA21300), and all patients signed the informed consent. TCGA and TCIA databases were publicly available for research, thus ethical approval was not required.

### 2.2 Histopathological image features

The size of whole‐slide image made it difficult to recognize features, thus we first used the Openslide Python library ^22^ to crop 395 whole‐slide images into 342,086 sub‐images. We divided the 40×images into sub‐images of 1000 × 1000 pixels, and divided the 20× images into sub‐images of 500 × 500 pixels to get a same perspective. Then we re‐sized the 500 × 500 pixel sub‐images to 1000 × 1000 pixels for further analysis. According to previous studies,^19^, ^23^ we excluded the sub‐images with less information, which had more than 50% white background. To decrease the computational cost, 20 sub‐images were randomly selected from the remaining images.^14^, ^19^ An eligible sub‐image contained about 500 cells, and each TMA sample contained about 1500 cells to extract the objective cellular attributes.

Image feature extraction was performed on CellProfiler (https://cellprofiler.org/).^24^ We first applied the “UnmixColors” module to separate the hematoxylin and eosin staining of images, then used image processing modules to convert color images to grayscale and correct the illumination. The next step was to use object identification modules to identify the nuclei and cell bodies for feature measurement. As previous reported,^13^ we extracted 10 aspects of image features by built‐in modules of CellProfiler, including Correlation, Image Area Occupied, Image Granularity, Image Intensity, Image Quality, Object Intensity, Object Neighbors, Object Radial Distribution, Object Size Shape, and Texture (Table S1). Unlike general pathological characteristics such as atypia and mitotic activity, these image features cannot be examined by microscopy, but are able to characterize the microscopic size, shape, and distribution of pixel intensity. For example, “Image Area Occupied” estimates the total area of an image, while “Object Size Shape” measures several cell‐level features, such as perimeter, eccentricity, and Zernike shape features. “Granularity” refers to size measurement of image texture by using enlarged structure elements to match the texture. After selection, 593 image features were eligible for analyses, and the average values of 20 sub‐images were calculated for each slide. When the patients had multiple slides, the average values of multiple slides were further calculated.

### 2.3 Classification of somatic mutations and molecular subtypes

In this part, we aimed to estimate the ability of histopathological image features in classifying mutations and subtypes of HNSCC. Five common somatic mutations (*TP53*, *CDKN2A*, *NOTCH1*, *NSD1*, and *PIK3CA*), transcriptional subtypes (atypical, basal, classical, and mesenchymal) and methylation subtypes (normal‐like, hypomethylated, hypermethylated, and CpG island hypermethylated) were involved. Due to the small number of samples with molecular feature information, a fivefold cross‐validation was conducted to obtain a stable model. Patients were randomly divided into 5 parts, 4/5 of which were regarded as the training set and 1/5 as the validation set. We calculated the average area under curve (AUC) of receiver operating characteristic curve (ROC) across five iterations in validation set. In each iteration, we first used the least absolute shrinkage and selection operator (LASSO)‐logistic regression for features reduction and selection.^25^ Next, three machine‐learning algorithms, the logistic regression (LR),^26^ random forest (RF)^27^ and support vector machine (SVM) ^28^ were trained to predict these variables (mutations and subtypes) using selected image features. The RF with 1000 decision trees was performed by R package “randomForest”.

### 2.4 Features selection and enrichment analysis

Using createDataPartition function of R package “caret”, the TCGA patients were randomly separated into training set and test set (7:3 ratio) based on the mortality. In training set, we utilized the LASSO‐Cox regression and SVM‐recursive feature elimination (SVM‐RFE) algorithm^29^ to select features subset with higher prediction accuracy for overall survival (OS), and avoid overfitting when the number of features was large. The overlapping features in the results were further included in subsequent analyses. Gene set enrichment analysis (GSEA) was applied to find enriched Kyoto Encyclopedia of Genes and Genomes (KEGG) pathways in high‐value group or low‐value group of image features.^30^ The *p* < 0.05 and false discovery rate (FDR) q<0.25 was statistically significant.^31^

### 2.5 Gene co‐expression network analysis

To better investigate the underlying biological mechanisms of histopathological image features, we performed weighted gene co‐expression network analysis (WGCNA) to find gene expression modules of training set.^32^ Briefly, we first used Pearson correlation analysis to estimate the relation between each gene pair. The adjacency matrix was calculated by weighted correlation between genes. We decreased the similarity of co‐expression to a power of β=8 (scale free R^2^=0.95) to ensure a scale‐free network. Then the topological overlap matrix (TOM) was transformed from adjacency matrix to reduce false correlation, which was utilized in hierarchical clustering analysis to identify gene modules. Modules were defined as clusters of highly absolute or positively correlated genes with high topological overlap. The module eigengene (ME) was the first principal component, which was the representative of module to explain the maximum variation of expression level. We estimated the correlation between MEs and image features to identify the key module. Finally, we conducted Gene Ontology (GO) enrichment analysis of key module via Metascape (http://metascape.org).

### 2.6 Construction of integrative prognostic model

Based on selected histopathological image features, we used RF algorithm and fivefold cross‐validation to build a prediction model for OS in training set, and further estimated it robustness in test set and external validation set. The analyses were performed using R package “randomForestSRC.” Then we defined the risk score assessed by this model as histopathological risk score (HRS). Patients were regarded as high‐risk and low‐risk groups by median HRS. Survival outcomes were showed in Kaplan–Meier survival curve and compared by log‐rank test. The hazard ratio (HR) and 95% confidence interval (CI) were calculated by Cox regression analysis.

According to the key module that most related to image features in WGCNA, we combined image features and expressed genes of module to establish an integrative prognostic model by RF algorithm. Similarly, the risk score calculated by this model was histopathological transcriptomics risk score (HTRS). Predictive performance of model was estimated in training and test sets. Furthermore, we conducted multivariate Cox analysis in training set to explore whether HTRS was independent of other prognostic factors, and formulated a nomogram. The calibration curves were applied to evaluate the goodness‐of‐fit between nomogram‐predicted OS and observed OS. Finally, the decision curve analysis was performed to compare the net benefits of models.^33^ Statistical analyses were conducted with R version 3.6.1.

## 3 RESULTS

### 3.1 Classification ability of image features on tumor mutations and subtypes

This study included 212 patients from TCGA cohort and 126 patients from TMA cohort (Table 1). There were significant differences in age, tumor site, and TNM stage between the two cohorts. TCGA‐HNSCC patients had older average age of onset, and higher rates of oral tumors and advanced stage (*p* < 0.001). The *TP53*, *CDKN2A*, *NOTCH1*, *NSD1*, and *PIK3CA* were most common somatic mutations in HNSCC.^9^, ^10^, ^11^ The transcriptional subtypes of HNSCC were classified based on gene expression profile, and related to different gene alterations.^9^ Furthermore, the methylation subtypes were strongly associated with mutations and transcriptional subtypes.^9^ Therefore, subtype classification may be an important step toward the biological processes research for HNSCC.

**TABLE 1:** Clinical and molecular characteristics of patients

Characteristic	TCGA‐HNSCC		*p* value^a^	TMA‐HNSCC	*p* value^b^
	Training set (%)	Test set (%)		Validation set (%)	
Sample size	149	63	–	126	–
Age: mean±SD	62.1 ± 12.8	61.9 ± 9.3	0.921	48.0 ± 11.0	<0.001
Gender					
Male	105 (70.5)	46 (73.0)		98 (77.8)	
Female	44 (29.5)	17 (27.0)	0.708	28 (22.2)	0.186
Tumor Site					
Larynx	38 (25.5)	18 (28.5)		16 (12.7)	
Oral cavity	93 (62.4)	38 (60.3)		23 (18.3)	
Pharynx	18 (12.1)	7 (11.1)	0.894	87 (69.0)	<0.001
TNM stage					
Ⅰ	8 (5.4)	3 (4.8)		15 (11.9)	
Ⅱ	26 (17.4)	9 (14.3)		54 (42.9)	
Ⅲ	24 (16.1)	9 (14.3)		37 (29.4)	
Ⅳ	91 (61.0)	42 (66.7)	0.893	20 (15.9)	<0.001
Tumor grade					
G1	16 (10.7)	5 (7.9)		NA	
G2	99 (66.4)	43 (68.3)			
G3	34 (22.8)	15 (23.8)	0.822		‐
Transcriptional subtype					
Basal	45 (30.2)	15 (23.8)		NA	
Mesenchymal	36 (24.2)	16 (25.4)			
Atypical	32 (21.5)	13 (20.6)			
Classical	21 (14.1)	11 (17.5)			
NA	15 (10.1)	8 (12.7)	0.803		‐
Methylation subtype					
Normal‐like	39 (26.1)	17 (27.0)		NA	
Hypo‐methylated	13 (8.7)	11 (17.5)			
Hyper‐methylated	56 (37.6)	19 (30.2)			
CpG island hyper‐methylated	26 (17.4)	8 (12.7)			
NA	15 (10.1)	8 (12.7)	0.228		‐

^a^ *p* value for comparison between training set and test set;

^b^ *p* value for comparison between TCGA and TMA cohorts.

To represent the clinical value of histopathological image features in HNSCC, we combined image features with three machine‐learning approaches (LR, RF, and SVM) to classify these mutations and subtypes in TCGA‐HNSCC patients (Table 2). Compared to LR and SVM, the RF achieved higher accuracy for predicting *TP53* (AUC = 0.930), *CDKN2A* (AUC = 0.913), *NOTCH1* (AUC = 0.903), *NSD1* (AUC = 0.828), *PIK3CA* (AUC = 0.871), basal (AUC = 0.954), mesenchymal (AUC = 0.930), atypical (AUC = 0.905), classical (AUC = 0.864), normal‐like (AUC = 0.942), hypomethylated (AUC = 0.881), hypermethylated (AUC = 0.968), and CpG island hypermethylated (AUC = 0.911). These results indicated that histopathological image features could classify these somatic mutations and molecular subtypes through the random forest algorithm.

**TABLE 2:** Area under ROC curve of machine‐learning methods in predicting mutations and subtypes

Characteristic	Logistic regression	Random forest	Support vector machine
Somatic mutation			
*TP53*	0.715	0.930	0.885
*CDKN2A*	0.689	0.913	0.816
*NOTCH1*	0.697	0.903	0.825
*NSD1*	0.723	0.828	0.815
*PIK3CA*	0.726	0.871	0.827
Transcriptional subtype			
Basal	0.726	0.954	0.845
Mesenchymal	0.783	0.930	0.864
Atypical	0.723	0.905	0.862
Classical	0.592	0.864	0.733
Methylation subtype			
Normal‐like	0.680	0.942	0.835
Hypo‐methylated	0.781	0.881	0.814
Hyper‐methylated	0.740	0.968	0.859
CpG island hyper‐methylated	0.732	0.911	0.844

### 3.2 Prognostic image features and enriched gene pathways

**FIGURE 2:**
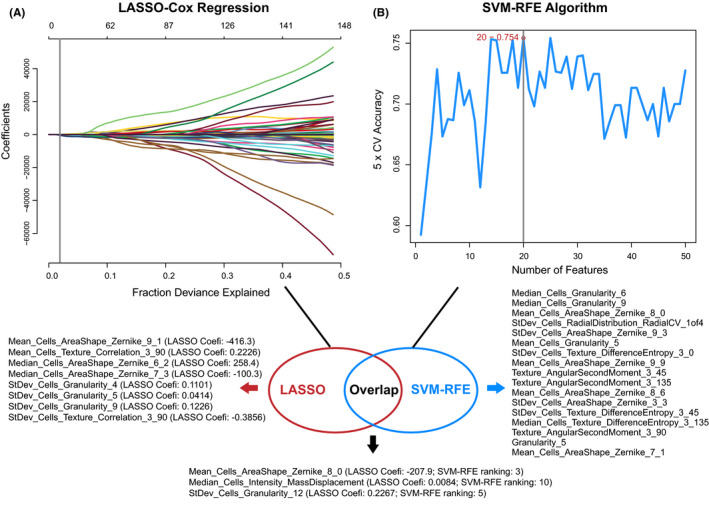
Selection of histopathological image features with significant prognostic value in the training set. (A) The least absolute shrinkage and selection operator (LASSO) identified 11 survival‐associated features. Coefi, coefficient. (B) The support vector machine‐recursive feature elimination (SVM‐RFE) selected 20 prognostic features (listed by ranking). Three image features were significant in two selection methods

The TCGA‐HNSCC cohort was divided into training set (*n* = 149) and test set (*n* = 63), which had no significant difference in patient's characteristics (Table 1). The prognostic value of 593 histopathological image features was estimated by LASSO and SVM‐RFE algorithms to identify which features were related to OS of training set. LASSO (L1 regularization) can shrink the regression coefficients of irrelevant features to zero, and thus select a small subset of features with non‐zero coefficients. The positive LASSO coefficient indicated that the higher feature was associated with poor prognosis, while the negative coefficient was in reverse. SVM‐RFE algorithm can rank each feature according to the weight magnitude and remove the lowest ranked feature, then use the remaining features for the next iteration, and finally determine the optimal number of features. We calculated the mean rankings of features depending on fivefold cross‐validation. The results of LASSO showed 11 survival associated features, and SVM‐RFE selected 20 features with the highest rankings, which both contained three image features, including Mean_Cells_AreaShape_Zernike_8_0, Median_Cells_Intensity_MassDisplacement, and StDev_Cells_Granularity_12 (Figure 2). The LASSO coefficients meant that higher Mean_Cells_AreaShape_Zernike_8_0 was associated with better survival, while the other two were unfavorable prognostic factors. The SVM‐RFE rankings revealed that Mean_Cells_AreaShape_Zernike_8_0 was more relevant to OS than the other two features (Figure 2).

**FIGURE 3:**
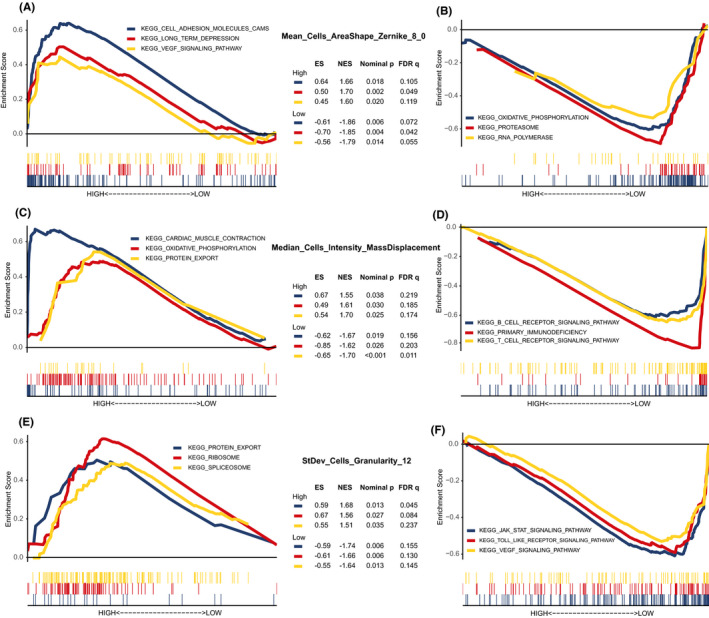
The enriched signaling pathways analyzed by gene set enrichment analysis in the training set. (A,B) Three representative pathways in groups with higher or lower Mean_Cells_AreaShape_Zernike_8_0. (C,D) Three representative pathways enriched in patients with high‐value or low‐value Median_Cells_Intensity_MassDisplacement. (E,F) Three representative pathways in high‐ or low‐StDev_Cells_Granularity_12 groups. ES, enrichment score; NES, normalized enrichment score; FDR, false discovery rate

Afterward, in the training set, we performed GSEA to assess the differences in enriched KEGG pathways between low‐value and high‐value groups (Figure 3). Most of enriched pathways were common dysregulated pathways in cancer, such as the vascular endothelial growth factor (VEGF), oxidative phosphorylation, proteasome, and spliceosome. Some others were related to immunity such as the B‐cell receptor (BCR), T‐cell receptor (TCR), and Toll‐like receptor. Moreover, we found that majority of the tumor development related pathways were enriched in the poor prognosis group, while most of the immune related pathways were enriched in the favorable prognosis group.

### 3.3 Gene modules correlated with prognostic image features

**FIGURE 4:**
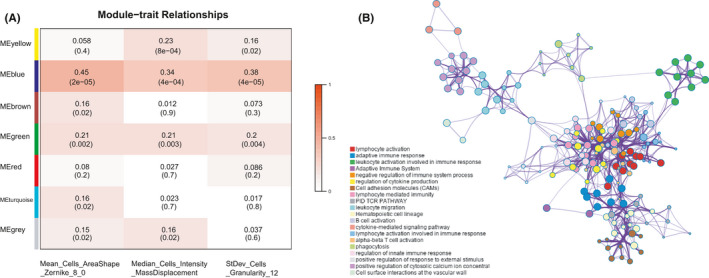
Identification and enrichment analysis of correlated gene modules in the training set. (A) Relationships between module eigengenes and histopathological image features. The blue module was most significant. (B) Enrichment network of blue module genes by Metascape. A circle node represented a term, its size depended on the number of input genes, and node color reflected the cluster identity. The most significantly enriched term was used to describe each cluster (see legend on left)

The WGCNA was utilized to reveal co‐expression networks and identify gene modules highly related to image features in the training set. The top 25% genes (4939 genes) with the largest variance were included. Seven distinct co‐expression modules were identified through the hierarchical clustering dendrogram. Next, we applied the correlation analysis to assess the relationship between the seven MEs and image traits (Figure 4A). The blue module (256 genes) had strongest correlation with Mean_Cells_AreaShape_Zernike_8_0 (|r| = 0.45), Median_Cells_Intensity_MassDisplacement (|r| = 0.34), and StDev_Cells_Granularity_12 (|r| = 0.38). Therefore, the blue module was considered for subsequent analyses, which might provide more accurate indication for histopathological image features.

Then we performed the functional annotation in GO enrichment analysis to explain the biological mechanisms related to blue module. The blue module genes were mainly enriched in categories of lymphocyte activation, adaptive immune response, leukocyte activation involved in immune response, and regulation of cytokine production (Figure 4B). It indicated that these genes were potentially associated with immune function, the tumor immunology has been a research focus in oncology and played an important role in occurrence and progression of tumors.

### 3.4 Prognostic value of histopathological transcriptomics risk score

**FIGURE 5:**
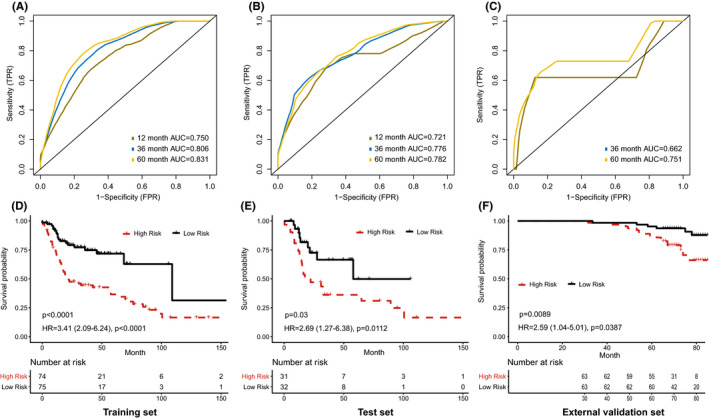
Prognostic model based on histopathological image features. (A‐C) The area under receiver operating characteristic curve (AUC) of the model for predicting overall survival in training, test, and external validation sets. (D‐F) Kaplan‐Meier survival curves of high‐risk and low‐risk groups in training, test, and external validation sets

We first established the histopathological model by the above three image features in the training set, and further assessed the stability of model in the test set and external validation set. The AUC for 5‐year OS was 0.831 in training set, 0.782 in test set, and 0.751 in external validation set (Figure 5A–C). Moreover, we obtained a histopathological risk score (HRS) from the model, and divided patients into high‐risk and low‐risk groups according to the median HRS. The log‐rank tests and Cox analyses indicated that high‐HRS patients had a significantly higher risk of death in the training set (HR = 3.41, 95%CI: 2.09–6.24, *p* < 0.001; Figure 5D), test set (HR = 2.69, 95%CI: 1.27–6.38, *p* = 0.011; Figure 5E) and external validation set (HR = 2.59, 95%CI: 1.04–5.01, *p* = 0.039; Figure 5F).

**FIGURE 6:**
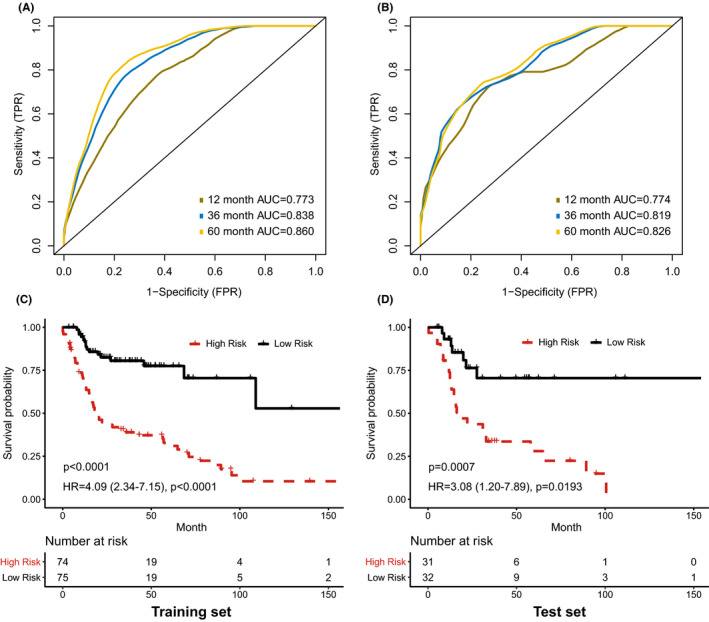
Prognostic model integrating histopathological image features with blue module genes. (A, B) The area under receiver operating characteristic curve (AUC) of the model for predicting overall survival in training and test sets. (C, D) Kaplan‐Meier survival curves of high‐risk and low‐risk groups in training and test sets

In addition, we built an integrative prognostic model with three image features and gene expression data of blue module. The integrative model achieved higher AUCs for predicting 1‐year, 3‐year, and 5‐year OS than the histopathological model in both training and test sets (Figure 6A,B). We called the risk score estimated by the integrative model as histopathological transcriptomics risk score (HTRS). The differences of survival outcomes between high‐HTRS and low‐HTRS groups were significant in the training set (HR = 4.09, 95%CI: 2.34–7.15, *p* < 0.001; Figure 6C) and test set (HR = 3.08, 95%CI: 1.20–7.89, *p* = 0.019; Figure 6D).

### 3.5 Nomogram establishment and evaluation

We applied univariate Cox analysis to show the prognostic value of clinical and molecular features in patients of training set (Table 3). The age at initial diagnosis (*p* = 0.027), TNM stage (Ⅰ vs. Ⅳ, *p* = 0.011) and HTRS (*p* < 0.001) were significantly associated with OS. The histological grade (G2) and transcriptional subtype (classical) might predict a worse prognosis, but the significance was weak. Furthermore, the prognostic features (*p* < 0.10) were enrolled in multivariate Cox analysis, which suggested that HTRS was an independent prognostic biomarker of OS (HR = 5.17, 95%CI: 2.82–9.41, *p* < 0.001).

**TABLE 3:** Univariate and multivariate Cox analyses in the training set

Variable	Category	Univariate analysis		Multivariate analysis	
		HR (95%CI)	*p* value	HR (95%CI)	*p* value
Age	Continuous	1.03 (1.00–1.04)	0.027	1.03 (1.01–1.05)	0.006
Gender	Female vs. Male	0.89 (0.54–1.44)	0.625	–	–
Tumor site	Oral cavity vs. Larynx	1.13 (0.67–1.90)	0.660	–	–
	Oral cavity vs. Pharynx	1.19 (0.56–2.55)	0.649	–	–
TNM stage	Ⅰ vs. Ⅱ	2.12 (0.75–6.01)	0.158	2.05 (0.72–5.82)	0.178
	Ⅰ vs. Ⅲ	2.39 (0.85–6.75)	0.099	2.30 (0.82–6.59)	0.116
	Ⅰ vs. Ⅳ	3.60 (1.33–9.72)	0.011	2.38 (1.15–5.13)	0.026
Tumor grade	G1 vs. G2	1.79 (0.93–3.44)	0.082	2.23 (0.99–5.00)	0.052
	G1 vs. G3	1.34 (0.65–2.77)	0.434	1.69 (0.71–4.06)	0.239
Transcriptional subtype	Classical vs. Atypical	0.55 (0.29–1.02)	0.058	1.05 (0.54–2.01)	0.896
	Classical vs. Basal	0.61 (0.34–1.08)	0.087	1.07 (0.58–1.98)	0.832
	Classical vs. Mesenchymal	0.67 (0.38–1.18)	0.162	1.30 (0.72–2.36)	0.391
HTRS	Low‐risk vs. High‐risk	4.09 (2.34–7.15)	<0.001	5.17 (2.82–9.41)	<0.001

Abbreviations: CI, confidence interval; HR, hazard ratio; HTRS, histopathological transcriptomics risk score.

**FIGURE 7:**
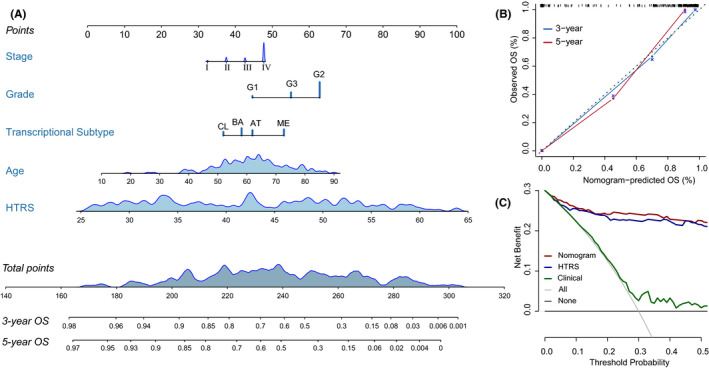
Nomogram construction and evaluation in the training set. (A) Nomogram for predicting the 3‐year and 5‐year overall survival (OS). (B) Calibration curves indicated the agreement between nomogram predicted and observed OS. (C) Decision curve analyses. The gray horizontal line represented net benefit of no intervention, while gray oblique line represented net benefit of intervening all patients. Compared with all‐treat scheme, non‐treat scheme, and clinical model, the nomogram and histopathological transcriptomics risk score (HTRS) had higher net benefit across the range of >10% in threshold probability

Based on multivariate Cox analysis, we generated a nomogram by combining HTRS and other prognostic features to predict 3 year and 5 year OS for patients in training set (Figure 7A). The Harrell's concordance index (C‐index) of nomogram was 0.768 (95%CI: 0.715–0.820). Moreover, the calibration curves reflected the good predictive performance of nomogram compared to an ideal model (Figure 7B). In the decision curves, the clinical model integrated age, stage, grade, and transcriptional subtypes. The HTRS showed a better net benefit than clinical model, and the nomogram achieved the highest net benefit across the most of threshold probability ranges (Figure 7C).

## 4 DISCUSSION

The “sub‐visual” histopathological image features that hardly be visually discriminated by pathologists probably offer a more quantitative measurement of cellular morphology, and help for better modeling of diagnosis and prognosis.^17^ Recent studies showed substantial interest in investigating the relationship between histopathology and omics data, and incorporating them for improved prediction of cancer development and patients’ outcomes.^18^, ^19^, ^20^, ^21^ In this study, we first used various machine‐learning classifiers to distinguish tumor mutations and subtypes by histopathological image features of HNSCC. Then we selected image features with significant prognostic value to form a histopathological prognostic model, and further identified correlated gene co‐expression module to establish an integrative prognostic model combining these two kinds of data. The results showed that the integrative model achieved outstanding performance of OS prediction. The histopathological transcriptomics risk score (HTRS) generated by this model was an independent prognostic factor. Additionally, the benefit of nomogram including HTRS significantly outperformed that of clinical model, which may facilitate the personalizing cancer management.

Genomics characterization at the transcriptional level divided HNSCC into four molecular categories with distinct patterns of gene dysregulation and biologic basis.^9^, ^34^, ^35^ Subtype 1 (basal) cancers were similar to basal subtype of lung SCC, which had high expression of *COL17A1*, *TGFA*, *EGFR*, and *TP63*.^34^ Subtype 2 (mesenchymal) showed over‐expression of genes associated with the epithelial‐to‐mesenchymal transition and elevated mutation of innate immunity genes.^9^, ^34^ Subtype 3 (atypical) tumors lacked *EGFR* amplification or 9p deletion, but displayed high positive rate of HPV and expression of *CDKN2A*, *LIG1*, and *RPA2*.^9^, ^35^ Finally, subtype 4 (classical) was characterized by heaviest smoking history and over‐expressed oxidative stress genes related to tobacco exposure (e.g., *NFE2L2* and *KEAP1*).^9^, ^34^ Differences of survival results were also found, basal subtype patients had significantly shorter recurrence‐free survival (RFS).^35^ Another study suggested no association between RFS and transcriptional subtypes, while for HPV^−^ patients, atypical cancers showed a higher risk of recurrence.^34^ The study found the worst OS outcome in classical subtype of laryngeal SCC, and higher risk of lymph node metastasis in mesenchymal subtype of oral SCC.^36^ In our study, the classical subtype revealed a trend of poorer OS, however, the p values were not enough statistically significant. The difference of results was probably due to the heterogeneity of tumor site and sample size in these studies. Therefore, the correlation between clinical outcomes and transcriptional subtypes still needs further validation in larger cohorts.

Aberrant DNA hypermethylation was considered to be involved in carcinogenesis and progression, such as suppressing the transcription of tumor suppressor genes and causing chromosomal instability.^37^ The hypomethylated subtype of HNSCC was associated with *NSD1* mutation, wild type *NOTCH1*, atypical, and classical subtypes.^9^ Conversely, hypermethylation and CpG island hypermethylation were more common in oral site tumors, basal, and mesenchymal subtypes.^9^ Although these gene expression and methylation signatures are not ready for clinical use, they provide new perspectives of HNSCC and has potential in final clinical application.^38^ In the present study, the histopathological image features showed a good capability to classify these subtypes and mutations. Compared with the previous study using CT radiomics features and LASSO to predict transcriptional subtypes and mutations,^39^ we combined LASSO with RF or SVM algorithms to build a more effective prediction model based on histopathological images. Therefore, histopathological image features analysis might serve as a convenient and low‐cost alternative strategy to predict the molecular subtypes and common mutations in HNSCC patients.

Afterward, we focused on the prognostic role of histopathological image features. Different from previous studies,^13^, ^14^, ^20^ we combined LASSO and SVM to obtain a more precise estimation. The Zernike, Displacement, and Granularity features were most significant, which indicated that properties of cellular morphology, intensity, and texture were related to survival results. Yu et al.^13^ also identified the prognostic value of Zernike features in lung cancer, which was consistent with our observation. The histopathological model based on three image features retained it robustness of survival prediction in the training set and test set. However, the tissue composition and proportion of the TMA cohort were not exactly the same with the TCGA cohort, which could lead to a worse performance in the external validation set. Furthermore, in the training and test sets, the integrative model of image features and transcriptomics data improved prediction accuracy than histopathological model and clinical factors. Our results suggested that the histopathology‐transcriptomics fusion may provide additional prognostic ability for patients whose survival outcomes were not well predicted by conventional clinical predictors. The nomogram showed a paradigm of prognostic strategy that incorporated the HTRS, transcriptional subtypes, and clinical variables. High‐risk patients may benefit from more aggressive treatments and strict follow‐up, while low‐risk patients should avoid excessive therapies.

For functional annotation, KEGG and GO enrichment analyses were performed to reveal the underlying biological processes. We found that they were enriched in immune function, such as BCR signaling pathway, TCR signaling pathway, lymphocyte activation, and adaptive immune response. The T cell‐mediated immune response has been widely researched in solid tumors, and applied in immunotherapies such as checkpoint inhibitors.^40^ The BCR signaling mainly influences the survival and growth of B‐cell leukemia or lymphoma cells, while the tumor‐infiltrating B cells were reported to promote tumor growth in SCC and pancreatic cancer.^41^, ^42^ Previous studies also showed the association between immune related pathways and morphological features of lymphocytes and tumor cells.^43^, ^44^ Moreover, we noticed the enrichment of VEGF signaling pathway in Zernike shape and Granularity features. This pathway is a key regulator of vasculogenesis and angiogenesis, which is aberrant in most tumors and correlates with vascular density, cell proliferation, invasiveness, metastases, and prognosis.^45^ These results may contribute to understand the molecular mechanisms regarding the morphological features of tumor cells.

Several limitations of our study should be noticed. First, this study had small sample size, because patients with matched histopathological images and genetic data were limited in TCGA database. The HPV status and treatment information were not available in most patients, which may be confounding factors affecting prognosis. Second, we externally validated the histopathological prediction model in the TMA cohort. However, such external validation was not conducted for the histopathological transcriptomics model due to the lack of transcriptomics data. Moreover, the representative tumor regions of TMA were analyzed in this study, while pathologists routinely utilized depth information of multiple slides and microscopic views. Therefore, the performance of prediction model in clinical application remains to be investigated. In this study, we randomly selected sub‐images for analysis. Using more whole‐slide images and more cells can further reduce potential bias and improve the rigorousness of research. The image features were calculated from the average value of sub‐images in our study, and the future research could more strictly determine the feature values. Finally, although the correlation analysis showed related biological processes, the key regulators for cellular morphology are still under exploration. Therefore, further large‐scale or experimental studies should be performed to confirm the clinical utility and molecular mechanisms of histopathological image features.

## 5 CONCLUSIONS

Our analyses suggested that histopathological image features were promising biomarkers for predicting genetic mutations, molecular subtypes and overall survival in HNSCC. Additionally, the integration of image features and gene expression data had potential for improving prognosis prediction. The proposed HTRS and nomogram provided prognostic estimation, and may contribute to the risk stratification and personalized treatment of HNSCC patients. However, large‐scale studies including more images and genetic data are still necessary to further verify the performance of our models.

## CONFLICT OF INTEREST

The authors declare no conflict of interest.

## ETHICAL APPROVAL STATEMENT

For tissue microarrays from Shanghai Outdo Biotech Company, the ethical approval was obtained from the National Human Genetic Resources Sharing Service Platform (2005DKA21300), and all patients signed the informed consent. TCGA and TCIA databases were publicly available for research, thus ethical approval was not required.

## Supporting information

Table S1

## Data Availability

The data that support the findings of this study are available upon reasonable request from the corresponding author.
