# Supplementary material for: Histopathological image and gene expression pattern analysis for predicting molecular features and prognosis of head and neck squamous cell carcinoma
Source: Cancer Med. 2021 May 13;10(13):4615–28. doi: 10.1002/cam4.3965 (PMC8267162; doi:10.1002/cam4.3965)
Supplement: Supplementary file 1 — Table S1 [file CAM4-10-4615-s001.doc]

**Table S1. Description of histopathological image features calculated from CellProfiler** **software.**

| **Type of image feature** | **Image feature name** | **Description** |
| --- | --- | --- |
| Correlation | Correlation coefficient | The correlation between a pair of images I and J. Calculated as Pearson's correlation coefficient, for which the formula is covariance(I,J)/[std(I)× std(J)]. |
|  | Slope | The slope of the least-squares regression between a pair of images I and J. Calculated using the model A×I+B=J, where A is the slope. |
| Image Area Occupied | AreaOccupied | The total area occupied by the input objects/binary image. |
|  | Perimeter | The total length of the perimeter of the input objects/binary image. |
|  | TotalImageArea | The total pixel area of the image. |
| Image Granularity | Granularity | The module returns one measurement for each instance of the granularity spectrum. |
| Image Intensity | TotalIntensity | Sum of all pixel intensity values. |
|  | MeanIntensity, MedianIntensity | Mean and median of pixel intensity values. |
|  | StdIntensity, MADIntensity | Standard deviation and median absolute deviation (MAD) of pixel intensity values. The MAD is defined as the median(|xi - median(x)|). |
|  | MinIntensity, MaxIntensity | Minimum and maximum of pixel intensity values. |
|  | LowerQuartileIntensity | The intensity value of the pixel for which 25% of the pixels in the object have lower values. |
|  | UpperQuartileIntensity | The intensity value of the pixel for which 75% of the pixels in the object have lower values. |
|  | TotalArea | Number of pixels measured. |
| Image Quality | PercentMaximal | Percent of pixels at the maximum intensity value of the image. |
|  | PercentMinimal | Percent of pixels at the minimum intensity value of the image. |
|  | FocusScore | A measure of the intensity variance across image. |
|  | LocalFocusScore | A measure of the intensity variance between image parts. |
|  | Threshold | The automatically calculated threshold for each image for the thresholding method of choice. |
|  | PowerLogLogSlope | The slope of the log-log magnitude and power spectra. |
| Object Intensity | IntegratedIntensity | The sum of the pixel intensities within an object. |
|  | MeanIntensity | The average pixel intensity within an object. |
|  | StdIntensity | The standard deviation of the pixel intensities within an object. |
|  | MaxIntensity | The maximal pixel intensity within an object. |
|  | MinIntensity | The minimal pixel intensity within an object. |
|  | IntegratedIntensityEdge | The sum of the edge pixel intensities of an object. |
|  | MeanIntensityEdge | The average edge pixel intensity of an object. |
|  | StdIntensityEdge | The standard deviation of the edge pixel intensities of an object. |
|  | MaxIntensityEdge | The maximal edge pixel intensity of an object. |
|  | MinIntensityEdge | The minimal edge pixel intensity of an object. |
|  | MassDisplacement | The distance between the centers of gravity in the gray-level representation of the object and the binary representation of the object. |
|  | LowerQuartileIntensity | The intensity value of the pixel for which 25% of the pixels in the object have lower values. |
|  | MedianIntensity | The median intensity value within the object. |
|  | MADIntensity | The median absolute deviation (MAD) value of the intensities within the object. The MAD is defined as the median(|xi - median(x)|). |
|  | UpperQuartileIntensity | The intensity value of the pixel for which 75% of the pixels in the object have lower values. |
|  | Location_CenterMassIntensity_X, Location_CenterMassIntensity_Y | The (X,Y) coordinates of the intensity weighted centroid (= center of mass = first moment) of all pixels within the object. |
|  | Location_MaxIntensity_X, Location_MaxIntensity_Y | The (X,Y) coordinates of the pixel with the maximum intensity within the object. |
| Object Neighbors | NumberOfNeighbors | Number of neighbor objects |
|  | PercentTouching | Percent of the object's boundary pixels that touch neighbors, after the objects have been expanded to the specified distance. |
|  | FirstClosestObjectNumber | The index of the closest object. |
|  | FirstClosestDistance | The distance to the closest object. |
|  | SecondClosestObjectNumber | The index of the second closest object. |
|  | SecondClosestDistance | The distance to the second closest object. |
|  | AngleBetweenNeighbors | The angle formed with the object center as the vertex and the first and second closest object centers along the vectors. |
| Object Radial Distribution | MeanFrac | Mean fractional intensity at a given radius; calculated as fraction of total intensity normalized by fraction of pixels at a given radius. |
|  | FracAtD | Fraction of total stain in an object at a given radius. |
|  | RadialCV | Coefficient of variation of intensity within a ring, calculated over 8 slices. |
| Object Size Shape | Area | The actual number of pixels in the region. |
|  | Perimeter | The total number of pixels around the boundary of each region in the image. |
|  | FormFactor | Calculated as 4*π*Area/Perimeter2. Equals 1 for a perfectly circular object. |
|  | Eccentricity | The eccentricity is the ratio of the distance between the foci of the ellipse and its major axis length. The value is between 0 and 1. (0 and 1 are degenerate cases; an ellipse whose eccentricity is 0 is actually a circle, while an ellipse whose eccentricity is 1 is a line segment.) |
|  | Solidity | The proportion of the pixels in the convex hull that are also in the region. Also known as convexity. Computed as Area/ConvexArea. |
|  | Extent | The proportion of the pixels in the bounding box that are also in the region. Computed as the Area divided by the area of the bounding box. |
|  | EulerNumber | The number of objects in the region minus the number of holes in those objects, assuming 8-connectivity. |
|  | MajorAxisLength | The length (in pixels) of the major axis of the ellipse that has the same  normalized second central moments as the region. |
|  | MinorAxisLength | The length (in pixels) of the minor axis of the ellipse that has the same  normalized second central moments as the region. |
|  | Orientation | The angle (in degrees ranging from -90 to 90 degrees) between the x-axis and the major axis of the ellipse that has the same second-moments as the region. |
|  | Compactness | The mean squared distance of the object’s pixels from the centroid divided by the area. A filled circle will have a compactness of 1, with irregular objects or objects with holes having a value greater than 1. |
|  | MaximumRadius | The maximum distance of any pixel in the object to the closest pixel outside of the object. For skinny objects, this is 1/2 of the maximum width of the object. |
|  | MedianRadius | The median distance of any pixel in the object to the closest pixel outside of the object. |
|  | MeanRadius | The mean distance of any pixel in the object to the closest pixel outside of the object. |
|  | MinFeretDiameter, MaxFeretDiameter | The Feret diameter is the distance between two parallel lines tangent on either side of the object. The minimum and maximum Feret diameters are the smallest and largest possible diameters, rotating the calipers along all possible angles. |
|  | Zernike shape features | Measure shape by describing a binary object in a basis of Zernike polynomials, using the coefficients as features. Currently, Zernike polynomials from order 0 to order 9 are calculated, giving in total 30 measurements. |
| Texture | Haralick Features | Haralick texture features are derived from the co-occurrence matrix, which contains information about how image intensities in pixels with a certain position in relation to each other occur together. Thirteen features are calculated for the image by performing mathematical operations on the co-occurrence matrix: AngularSecondMoment, Contrast, Correlation, Variation, InverseDifferenceMoment, SumAverage, SumVariance, SumEntropy, Entropy, DifferenceVariance, DifferenceEntropy, InfoMeas1, InfoMeas2. Each measurement is suffixed with the direction of the offset used between pixels in the co-occurrence matrix: 0: Horizontal, 90: Vertical, 45: Diagonal, 135: Anti-diagonal. |
|  | Gabor "wavelet" features | These features are similar to wavelet features, and they are obtained by applying so-called Gabor filters to the image. The Gabor filters measure the frequency content in different orientations. The Gabor features detect correlated bands of intensities, for instance, images of Venetian blinds would have high scores in the horizontal orientation. |

Reference:

1. Carpenter AE, Jones TR. CellProfiler 2.2 Manual. Retrieved March, 2021, from https://cellprofiler.org/manuals.

2. Carpenter AE, Jones TR, Lamprecht MR, et al. CellProfiler: image analysis software for identifying and quantifying cell phenotypes. Genome Biol. 2006;7(10):R100.

3. Lamprecht MR, Sabatini DM, Carpenter AE. CellProfiler: free, versatile software for automated biological image analysis. Biotechniques. 2007;42(1):71-75.
